# Hemidiaphragmatic paralysis following costoclavicular versus supraclavicular brachial plexus block: a randomized controlled trial

**DOI:** 10.1038/s41598-021-97843-x

**Published:** 2021-09-21

**Authors:** Boohwi Hong, Soomin Lee, Chahyun Oh, Seyeon Park, Hyun Rhim, Kuhee Jeong, Woosuk Chung, Sunyeul Lee, ChaeSeong Lim, Yong-Sup Shin

**Affiliations:** 1 Department of Anesthesiology and Pain Medicine, Chungnam National University Hospital, 282 Munhwa-ro, Jung-gu, Daejeon, 35015 Korea; 2 Department of Anesthesiology and Pain Medicine, College of Medicine, Chungnam National University, Daejeon, Republic of Korea; 3 Department of Nursing, Chungnam National University, Daejeon, Republic of Korea

**Keywords:** Diagnosis, Risk factors, Clinical trials, Randomized controlled trials

## Abstract

Costoclavicular brachial plexus block is emerging as a promising infraclavicular approach performed just below the clavicle. However, there are relatively little data regarding the hemidiaphragmatic paralysis (HDP) compared to the commonly performed supraclavicular block. We hypothesized that the incidence of HDP in costoclavicular block is lower than supraclavicular block like classical infraclavicular approach. Eighty patients were randomly assigned to ultrasound-guided supraclavicular (group S) or costoclavicular (group C) block with 25 mL of local anesthetics (1:1 mixture of 1% lidocaine and 0.75% ropivacaine). The primary outcome was the incidence of HDP, defined as less than 20% of fractional change in the diaphragm thickness on ultrasound M-mode. Also, pulmonary function test and chest radiograph were assessed before and after the surgery. The incidence of HDP was 4/35 (11.4%) in the group C and 19/40 (47.5%) in the group S (risk difference, − 36%; 95% CI − 54 to − 17%; P = 0.002). The mean (SD) change of DTF values were 30.3% (44.0) and 56.9% (39.3) in the group C and S, respectively (difference in means, − 26.6%; 95% CI − 45.8 to − 7.4%; P = 0.007). The pulmonary function was more preserved in group C than in group S. The determined diagnostic cut off value of the diaphragm elevation on chest radiograph was 29 mm. Despite the very contiguous location of the two approaches around the clavicle, costoclavicular block can significantly reduce the risk of HDP compared with supraclavicular block.

## Introduction

The costoclavicular brachial plexus block is a recently introduced infraclavicular approach, and it is known to exhibit a rapid and reliable blockade^1–3^. In the costoclavicular space, the three cords of the brachial plexus are clustered together while each cord maintains a consistent relation to one another. Also this space is more superficial than the space where the classical infraclavicular approach targets. Costoclavicular block has shown to be a retrograde channel to the supraclavicular area, and it can involve the proximal branch of the brachial plexus, such as suprascapular nerve, which enables reliable analgesia for arthroscopic shoulder surgery^4^. Also, the catheter for continuous regional anesthesia can be introduced from the costoclavicular space to the corner pocket of the supraclavicular area allowing additional stability^5^. Because of these clinical and anatomical advantages, it is emerging as an attractive approach for brachial plexus block^6^.

Hemidiaphragmatic paralysis (HDP) is a common complication after brachial plexus block, especially when performed above the clavicle. The incidence of HDP is reported up to 70% in supraclavicular approach^7,8^. Even though well tolerated in healthy patients, it can be a critical issue in patients with marginal pulmonary function^9^. The close distance between the block site and the C3–C5 nerve root or the phrenic nerve is thought to be critical in the occurrence of HDP^10^. Because of the very contiguous location around the clavicle, costoclavicular approach may also have a high risk of HDP. In a study using computed tomography reconstruction, the contrast medium injected from the costoclavicular space was observed to reach the interscalene region at the level of primary trunk of brachial plexus^11^. Contrary to such concerns, previous studies reported the incidence of HDP in the costoclavicular approach to be comparable to the paracoracoid approach^12^. We have also reported retrospective data using chest radiography, suggesting a lower incidence of HDP in costoclavicular approach compared to the supraclavicular approach^13^.

However, only few randomized trials have compared the incidence of HDP after costoclavicular and supraclavicular block. As the supraclavicular approach is the most widely used approach, we compared costoclavicular block with supraclavicular block. We hypothesized that the incidence of HDP in costoclavicular block is lower than supraclavicular block. Also, the change of the pulmonary function according to the blockade was assessed to evaluate the actual impact of the blockade on patient.

## Methods

### Study design and subjects

This randomized, controlled, observer-blinded superiority trial was performed at the Chungnam National University Hospital, Republic of Korea and adhered to the tenets of the Declaration of Helsinki. This study was approved by the Chungnam National University Hospital’s Institutional Review Board (IRB #CNUH 2019-04-031-002) and written informed consent was obtained from all subjects participating in the trial. The trial was registered prior to patient enrollment at cris.nih.go.kr (KCT0004023, Principal investigator: Boohwi Hong, Date of registration: 31/05/2019).

Patients were evaluated for the eligibility of the study, and written informed consents were obtained from enrolled participants by the study team before surgery. The trial cohort consisted of aged 20–70 years with American Society of Anesthesiologists (ASA) physical status I or II scheduled for orthopaedic upper limb surgery (including elbow, forearm, hand, and wrist surgery) with regional anesthesia. The exclusion criteria included hypersensitivity to amide anesthetic, preexisting neuropathy on surgical limb, significant pulmonary disease, coagulopathy, sepsis, infection on block site, pregnancy, conversion to general anesthesia, and patient refusal. The significant pulmonary disease included all pulmonary morbidities in which the respiratory compromise was expected in the case of HDP.

Study data were collected and managed using REDCap (Research Electronic Data Capture) software hosted at Chungnam National University Hospital (http://redcap.cnuh.co.kr/). The REDCap is a secure, web-based platform designed to support capturing of data for research studies^14^. This manuscript adheres to the applicable CONSORT (Consolidated Standards of Reporting Trials) guidelines^15^.

### Randomization and minimization of bias

Patients were randomly assigned at a ratio of 1:1 to one of the two groups: the costoclavicular (group C) block and the supraclavicular (group S) block. Block randomization with size of 2 and 4 was performed on a website (www.randomization.com) with a random sequence generator. To conceal the allocation, the sequence was uploaded on the REDCap (http://redcap.cnuh.co.kr/), allowing access only to the researcher performing the assigned block. All other individuals who participated in the surgery, attending anesthesiologist, surgeon, nurses, and outcome assessor were blinded to the group assignment. For the study participants, however, complete blinding was not achievable due to the pain in the puncture site during the blockade.

### Anesthetic procedures

Patients were premedicated with 0.05 mg/kg of midazolam intravenously before entering the operating room. Standard ASA monitoring was applied before performing the block and maintained throughout the entire procedure. All blocks were performed under ultrasound guidance by a single experienced anesthesiologist (B.H) using an in-plane technique with MylabTM25 Gold (Esaote, Genova, Italy) and a linear probe (LA435: 6–18 MHz, Esaote). Each patient was administered 25 mL of local anesthetic, consisting of a 1:1 mixture of 1% lidocaine and 0.75% ropivacaine. All four terminal nerve (i.e. radial, median, ulnar, musculocutaneous) dermatomes including expected surgical field were assessed for surgical readiness. After confirming loss of pinprick sensation, the patient was allowed to proceed surgical preparation. The sedation was induced using dexmedetomidine (loading dose for 1 µg/kg over 10 min and maintenance dose for 0.2–0.5 µg/kg/h) and discontinued at the beginning of skin suture. Supplemental oxygen was administered prior to sedation at a rate of 5 L/min via simple facial mask. After about 30 min of observation in PACU and confirming alert mental status, the movement of diaphragm was assessed by ultrasonography. All measurements were recorded by the attending anesthesiologist.

### Costoclavicular block

**Figure 1:**
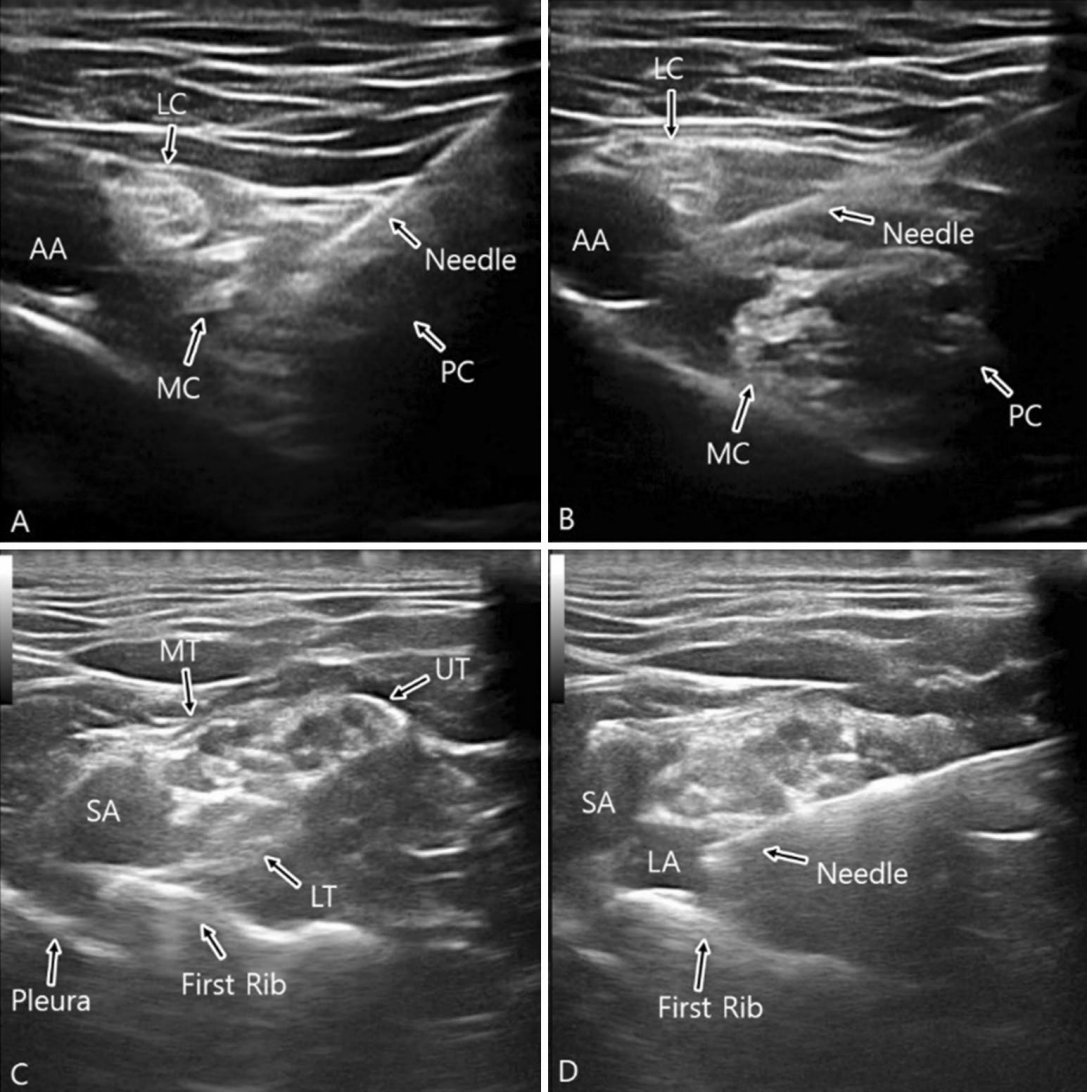
Sonographic view of costoclavicular (**A,B**) and supraclavicular (**C,D**) block.

In group C, patients were placed in a supine position with the surgical limb abducted at an angle of 90 degrees. A transverse scan was performed immediately below the midpoint of the clavicle and over the medial infraclavicular fossa. Maintaining the same position, the transducer was gently tilted cephalad to direct the ultrasound beam toward the costoclavicular space, defined as the space between the posterior surface of the clavicle and the second rib. The ultrasound image was optimised until all three cords of the brachial plexus were visualised lateral to the axillary artery. Using an in-plane technique and a lateral-to-medial direction, the block needle was advanced until its tip was located in the middle of the three cords. Initially, 15–20 mL of local anesthetic was injected between the medial and posterior cord. The needle was then slightly withdrawn and its tip was relocated adjacent to the lateral cord, and an additional 5–10 mL of anesthetic was injected^2,13^ (Fig. 1A,B).

### Supraclavicular block

In group S, patients were placed in a supine position, with a soft (jelly) pad placed behind the back and the head turned slightly to the contralateral side. A transverse scan was performed immediately above the clavicle. The transducer was gently tilted caudad to direct the ultrasound beam toward the first rib. The first target was the corner pocket (i.e., the intersection between the first rib and the subclavian artery). Subsequently, the needle was repositioned under direct vision and directed toward the neural cluster formed by the trunks and divisions of the brachial plexus. 5 to 10 mL of local anesthetic was injected into the corner pocket, followed by an injection of 15–20 mL between each trunk or division^13,16,17^ (Fig. 1C,D).

### Sonographic assessment of diaphragm

**Figure 2:**
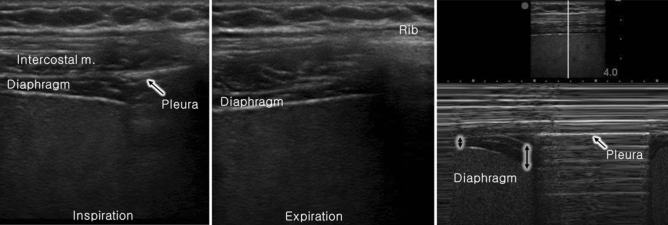
Diaphragm thickness fraction (DTF) measurement. DTF = thickness at end inspiration − thickness at end expiration/thickness at end expiration. Hemidiaphragmatic paralysis was defined as DTF < 20%.

Sonographic assessment of the diaphragmatic motion and contraction was performed before blockade and after surgery according to the “ABCDE” method described by Tsui et al.^18^. The “ABCDE” method is performed by placing the probe at the zone of apposition of anterior Axillary line, watching for Breathing (lung sliding), then moving Caudally to identify the Diaphragm for Evaluation. During full inspiration and breath holding at total lung capacity, thickening of the diaphragm observed under sonography indicates that the diaphragm is shortening and contracting. The diaphragm thickening fraction (DTF) is calculated as: (thickness at inspiration − thickness at expiration)/thickness at expiration (Fig. 2).

### Outcomes measures

In this study, the primary outcome was the incidence of HDP, defined as less than 20% of DTF on M-mode ultrasound^19^. Complete and partial hemidiaphragmatic paralysis were defined as less than 5% of DTF (or the paradoxical movement of diaphragm), and between 5 and 20% of DTF, respectively.

The change in pulmonary function after the block and the elevation of the hemidiaphragm on postoperative chest radiograph were assessed as secondary outcomes. The spirometry (CONTEC SP10 BP Spirometer, Healthcare4all Ltd, United Kingdom) was performed in sitting position to assess pulmonary function before the blockade and after the surgery in the post-anesthesia care unit. The third value of spirometry was obtained and recorded after the two preceding exercises. The elevation of the hemidiaphragm was determined by comparing its relative position to that on the contralateral side on pre- and postoperative chest radiographs.

These chest radiographs were taken as a part of routine clinical practice in our institution. The level of the hemidiaphragm was measured by drawing two lines parallel to the vertebral body that passed the highest points of each hemidiaphragm. The height of the hemidiaphragm was determined by adding or subtracting the vertical distance between these two lines on the two hemidiaphragms^13^. The diagnostic cut off value of hemidiaphragm elevation was determined based on the diagnosis of HDP by DTF. The outcome assessor (S.M., blinded to the group allocation) measured DTF, pulmonary function test, chest radiography values, and block related complication such as dyspnea, desaturation, and pneumothorax.

### Statistical analysis

The sample size of this study was decided based on pilot study and another previous study. According to Petrar et al.^20^, brachial plexus block approaching below the clavicle, in comparison to approaching above the clavicle, reduced the risk of HDP (34% vs 3%). In our unpublished data, HDP occurred in about 35% of the patients who received blockade via the supraclavicular approach. Based on the assumption that there is 30% of risk difference in the HDP incidence by group, each group should have 33 subjects to have a power of 90% and a risk of 5% for type I errors. Taken into account of the compensation for potential dropouts and data losses, we aimed to recruit 80 subjects.

All analyses were performed by per protocol analysis using R (version 4.0.2)^21^. Continuous variables were analyzed by the independent t-test (mean ± SD) or the Mann–Whitney *U* test (median [IQR]) depending on the results of Shapiro–Wilk tests. Categorical variables were analyzed using *χ*^2^ or Fisher’s exact (expected count < 5) and recorded as number (%). For the primary outcome, the absolute risk difference with its 95% confidence interval (CI) was calculated. A two-tailed P-value < 0.05 was considered statistically significant. For the repeated measured secondary outcome (pre, post, delta value), P-value < 0.016 was considered statistically significant using Bonferroni correction. Since there was no pre-defined diagnostic criterion of the diaphragm elevation on chest radiography for HDP, the cut off value was determined by receiver operating curve based on the diagnosis of HDP by DTF.

## Results

### Study participants

**Figure 3:**
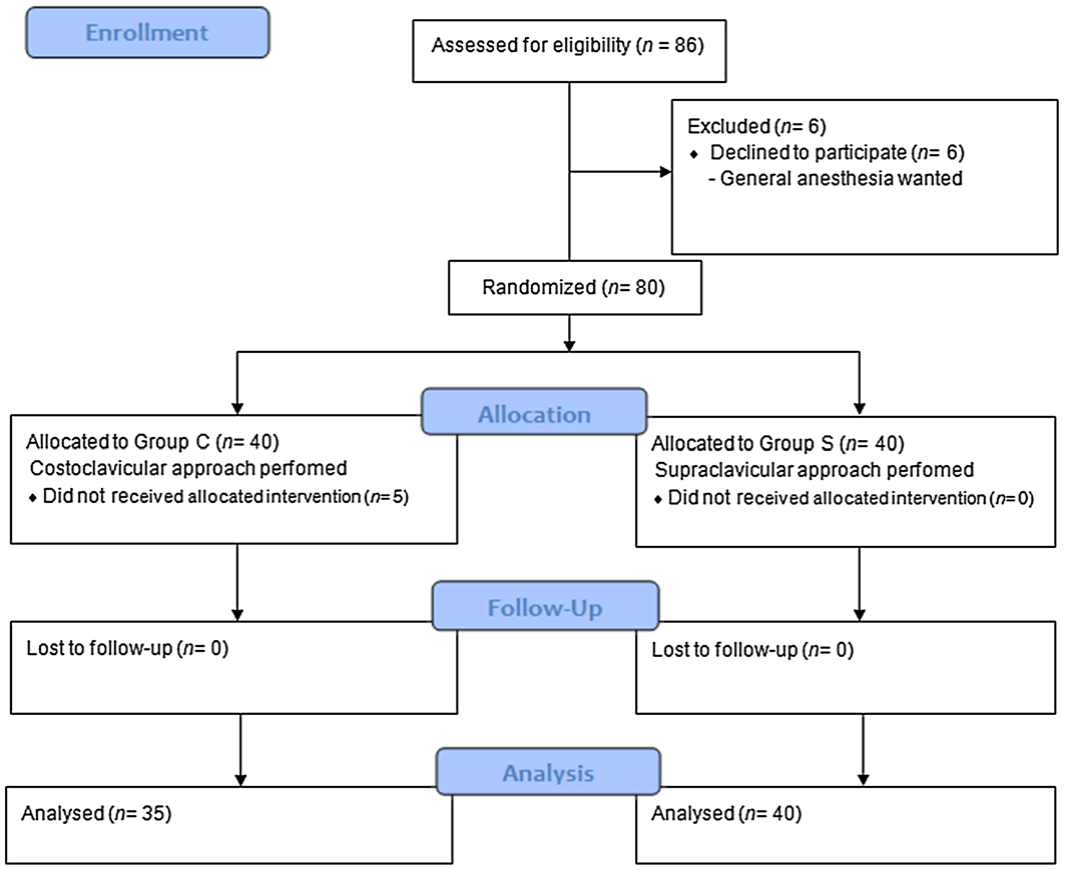
Consolidated standards of reporting trials (CONSORT) flow diagram.

From 31 May 2019 to 23 August 2019, 86 patients were assessed for eligibility, among which six refused to participate in the study, and were thus excluded. The remaining 80 patients were assigned either group S or group C. Five patients in group C unexpectedly received supraclavicular block and were excluded from the analysis. Of these five excluded patients, the location of the costoclavicular space was too deep to be optimally visualized which made it extremely difficult to clearly discriminate the cords. The Consolidated Standards of Reporting Trials (CONSORT) diagram is shown in Fig. 3. The demographic and clinical characteristics of the participants of both groups were comparable (Table 1). There was no failure of blockade (conversion to general anesthesia or additional infiltration of local anesthetics needed in the field) in all patients.

**Table 1:** Demographic and clinical characteristics.

	Group C (n = 35)	Group S (n = 40)	SMD
Female, *n* (%)	17 (48.6%)	19 (47.5%)	0.021
Age (year), median [IQR]	47.0 [41.0 to 61.5]	44.5 [35.5 to 58.0]	0.269
Height (cm), mean (SD)	164.8 (7.9)	165.8 (8.7)	0.118
Weight (kg), median [IQR]	61.0 [55.0 to 76.5]	63.3 [56.0 to 71.1]	0.110
BMI (kg/m^2^), median [IQR]	23.1 [21.5 to 26.3]	22.9 [20.8 to 25.4]	0.198
Operation side (Lt./Rt.)	15/20	19/21	0.093
Surgical site (elbow/forearm/hand/wrist)	4/3/13/15	1/1/11/27	0.590
Operation time (min), median [IQR]	56.0 [43.5;78.5]	57.5 [45.5;75.0]	0.026

*BMI* body mass index, *SMD* standardized mean difference.

### Primary outcome

The incidence of HDP was 4/35 (11.4%) in the group C and 19/40 (47.5%) in the group S (risk difference [RD], − 36%; 95% CI − 54 to − 17%; P = 0.002). Among these HDP cases, complete paralysis was 1/35 (2.9%) in the group C and 9/40 (22.5%) in the group S (RD, − 20%; 95% CI − 34 to − 6%; P = 0.031). The mean (SD) change of DTF values were 30.3 (44.0) % and 56.9 (39.3)% in the group C and S, respectively (difference in means, − 26.6%; 95% CI − 45.8 to − 7.4%; P = 0.007) (Table 2).

**Table 2:** Sonographic measurements of diaphragm and results of spirometry stratified by group.

	Group C (n = 35)	Group S (n = 40)	Effect size (95% CI)	p-value
**HDP, *n* (%)**	4 (11.4%)	19 (47.5%)	− 36 (− 54 to − 16)	0.002^a^
Complete , *n* (%)	1 (2.9%)	9 (22.5%)		
Partial, *n* (%)	3 (8.6%)	10 (25.0%)		
No paralysis, *n* (%)	31 (88.6%)	21 (52.5%)		
**Diaphragm thickness (mm)**				
Pre-block inspiration, median [IQR]	3.6 [2.9; 4.2]	4.0 [3.2; 4.8]	− 0.4 (− 1 to 0.3)	0.435^c^
Pre-block expiration, median [IQR]	1.8 [1.6; 2.5]	2.1 [1.7; 2.5]	− 0.3 (− 0.5 to 0.2)	0.555^c^
Post-block inspiration, median [IQR]	3.1 [2.7; 3.7]	2.5 [2.0; 3.2]	0.6 (0.15 to 1.1)	0.009^c^
Post-block expiration, median [IQR]	1.9 [1.6; 2.4]	2.0 [1.6; 2.2]	0.1 (− 0.3 to 0.2)	0.898^c^
Δ inspiration, median [IQR]	0.4 [ 0.0; 1.0]	1.4 [ 0.4; 1.9]	− 1 (− 1.3 to − 0.3)	0.012^c^
Δ expiration, median [IQR]	0.0 [− 0.2; 0.2]	0.0 [− 0.2; 0.2]	0 (− 0.2 to 0.1)	0.757^c^
**DTF (%)**				
Pre-block, median [IQR]	87.5 [63.8;115.1]	87.2 [64.5;112.6]	0.3 (− 16.3 to 24.5)	0.907^c^
Post-block, median [IQR]	64.0 [31.2;80.0]	21.8 [6.1;51.2]	42.2 (16.2 to 56.2)	0.001^c^
Δ DTF, mean (SD), 95% CI	30.3 (44.0), 12.3 to 480.2	56.9 (39.3), 38.3 to 750.5	− 30.5 (− 60.4 to − 11.3)	0.007^b^
**Pre-block**				
FVC (L), mean (SD)	3.2 (0.9)	3.3 (1.0)	− 0.2 (− 0.6 to − 0.3)	0.481^b^
FEV1 (L), mean (SD)	2.8 (0.8)	2.9 (0.9)	− 0.04 (− 0.4 to 0.3)	0.983^b^
PEFR (L/s), mean (SD)	6.7 (2.1)	6.1 (2.2)	0.5 (− 0.4 to 1.5)	0.275^b^
**Post-block**				
FVC (L), mean (SD)	3.1 (0.9)	2.8 (0.9)	0.3 (− 0.1 to 0.7)	0.122^b^
FEV1 (L), mean (SD)	2.6 (0.7)	2.3 (0.9)	0.3 (− 0.1 to 0.6)	0.144^b^
PEFR (L/s), mean (SD)	6.0 (2.1)	4.9 (2.3)	1.1 (0.2 to 2.1)	0.025^b^
Δ FVC (L), median [IQR]	0.1 [− 0.1; 0.3]	0.4 [0.2; 0.8]	− 0.3 (− 0.5 to − 0.2)	< 0.001^c^
Δ FEV1 (L), median [IQR]	0.2 [0.0, 0.3]	0.5 [0.2; 0.7]		
Δ PEFR (L/s), mean (SD)	0.7 (1.3)	1.2 (1.4)	− 0.6 (− 1.2 to 0.04)	0.069^b^
Δ FVC (%), mean (SD)	1.9 (10.6)	15.6 (16.8)	− 12.2 (− 20.3 to − 3.6)	< 0.001^b^
Δ FEV1 (%), mean (SD)	6.4 (9.2)	18.3 (17.7)	− 12.5 (− 19.1 to − 0.5)	< 0.001^b^
Δ PEFR (%), mean (SD)	8.5 (18.2)	20.4 (25.8)	− 11.4 (− 22.1 to 0.2)	0.002^b^

Effect size (risk difference for categorical variable, mean or median differences for continuous variables) are differences (Group C − Group S). The p-value < 0.016 was considered statistically significant using Bonferroni correction except the primary outcome.

*DTF* diaphragm thickning fraction, *HDP* hemidiaphragmatic paralysis (DTF < 20%), *FVC* forced vital capacity, *FEV1* forced expiratory volume in 1 s, *PEFR* peak expiratory flow rate, *Δ delta* (pre–post value), *CI* confidence interval.

^a^Fisher’s exact test.

^b^Independent t-test.

^c^Mann–Whitney *U* test.

### Secondary outcome

The mean reduction of spirometric values was significantly lower in group C in regards to all measures of the pulmonary function test, including forced vital capacity (FVC), forced expiratory volume in 1 s (FEV1), and peak expiratory flow rate (PEFR) (Table 2). The spirometric and sonographic measurments stratified by the occurrence of HDP is summarized in Supplementary Table S1.

**Figure 4:**
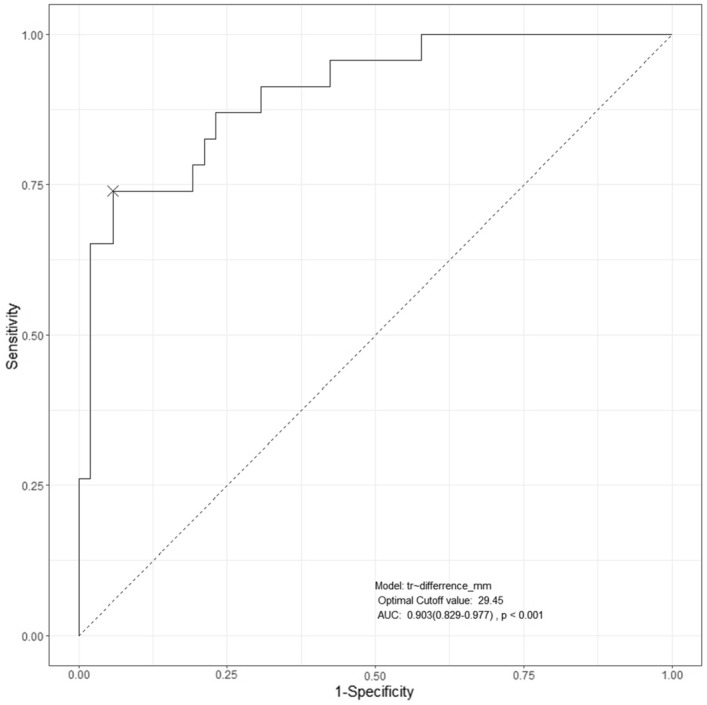
Receiver operating characteristic curves for elevation of the hemidiaphragm on chest radiographs. Optimal cut-off value: 29 mm (sensitivity 73.9%, specificity 94.2%) with an area under curve of 0.903 (95% CI 0.829 to 0.977; P < 0.001).

The median (IQR) change of diaphragm level on chest radiograph was 2.0 [− 2.8; 12.8] mm in the group C and 23.1 [3.7; 49.2] mm in the group S (Difference of medians, − 21.1 mm; 95% CI − 37.6 to − 11.5; P < 0.001). The determined diagnostic cut off value of the diaphragm elevation on chest radiograph was 29.45 mm (sensitivity 73.9%, specificity 94.2%) with an area under curve of 0.903 (95% CI 0.829 to 0.977; P < 0.001) (Fig. 4). With this cut off value for chest radiograph, the incidence of HDP was 3/35 (8.6%) in the group C and 18/40 (45.0%) in the group S (P = 0.001).

None of the other significant block-related complaints or complication such as dyspnea, desaturation, and pneumothorax were observed.

## Discussion

In this study, we evaluated the incidence of HDP after costoclavicular or supraclavicular block. Our findings suggest that costoclavicular block can reduce the risk of HDP compared to supraclavicular block. In addition, pulmonary function was more preserved in patients who received the costoclavicular block than those who received the supraclavicular block.

Since costoclavicular block is relatively a recent method, there are few data reporting its association with HDP^4,22^. In the meantime, our result seems consistent with other former studies which demonstrate reduced risk of HDP in the costoclavicular block. Aliste et al. reported 0 incidence of HDP in 22 cases of costoclavicular block^4^. In the study above, HDP was defined as “an absence of movement or a paradoxical movement of the diaphragm on ultrasound M-mode” using liver and spleen as acoustic window. Since it was an all or nothing criterion, it could only discriminate complete paralysis of diaphragm and may have missed any incomplete paralysis. Unfortunately, direct comparison with other studies using diaphragm excursion^23,24^, which discriminate complete and partial paralysis by 25% and 75% reduction of diaphragmatic movement, are limited due to the absence of results showing detailed sonographic measurements. Also, the volume of local anesthetic they used for blockade in their study was 20 mL, which also can be interpreted as a protective factor for HDP in comparison to our protocol. In a more recent study, Sivashanmugam et al.^22^ reported an incidence of HDP in 1 of 20 patients who received the costoclavicular block. Similar to the study by Aliste et al., they also utilized the ultrasound M-mode and 20 mL of local anesthetic. However, the subjects in their study only included the patients receiving surgeries on the right upper limb for easier assessment of the diaphragm through the liver’s acoustic window rather than the splenic window. They defined the HDP by at least 50% decrement in the diaphragm excursion.

The definition of HDP used in this study was based on the former study which assessed the change of the diaphragm thickness during maximal inspiration in 150 normal subjects^19^. The value of 20% we applied in terms of DTF, therefore, actually represents the lower limit of normal range. It is distinct from the definition used in the study by Aliste et al.^4^ and rather similar to the one used in the study by Sivashanmugam et al.^22^. According to Gottesman et al.^25^ − 35% to 5% change of DTF was noted in patients with HDP, and suggested same cutoff value of 20%. Since there was no pre-defined cut off value, less than 5% of DTF or paradoxical movement for complete paralysis was determined in our study itself. Additionally, we subdivided HDP cases as complete and partial paralysis and there was only one case of complete paralysis in group C compared to 9 cases in group S.

There are several studies reporting spirometric changes after brachial plexus blockade. Renes et al. reported at least 20% reduction of FEV1, FVC, and PEF in complete HDP cases^23^. This result is much greater than our measurements in HDP cases (median 24.8 and 16.5% of FEV1 and FVC, respectively) suggesting the presence of mixed HDP (partial and complete) in our current study. Another study reported 24.4 and 19.3% reduction of FEV1 and FVC respectively in supraclavicular block using the “corner pocket” method^24^. Although direct comparison is limited due to the discrepancy in the definition and proportion of HDP, the results are similar with our present study. On the other hand, a different study reported less than an 18% reduction of spirometric values in 15 cases of complete HDP after supraclavicular blockade^26^.

Phrenic nerve arises principally from the C4 root with less contribution from C3 and C5. It descends almost vertically on the anterior surface of the anterior scalene muscle, behind the pre-vertebral fascia. It may receive branches from various origins including the brachial plexus, spinal accessory nerve or the hypoglossal nerve. Additionally, about 30% of the phrenic nerve pathways are variants of the standard anatomy^27^. Also accessory phrenic nerve may arise from either the brachial plexus, nerve to subclavius, or the cervical plexus such as supraclavicular nerve and ansa cervicalis. It joins the phrenic nerve at varying levels around subclavian vessels^28^. Considering these factors, there is a possibility of partial block which causes insignificant effect on the pulmonary function^29^. If a small portion of local anesthetic barely reaches the phrenic nerve roots or bathes other contributing branches, partial phrenic nerve blockade may likely occur. This explanation can support the result of partial HDP (more than 5% and less than 20% of DTF) observed in our study.

The technique utilized for assessing the diaphragm in this study was different from other former studies. We adapted the “ABCDE” approach, a novel technique of diaphragmatic evaluation^18,30,31^. This technique is independent of hepatic or splenic acoustic window, and only requires the observation of diaphragmatic movement through intercostal muscle. Therefore, there are no limitations in assessing the left hemidiaphragm, which can often be troublesome due to the small splenic acoustic window or in case of the presence of an air-filled stomach^32,33^. It also allows the use of the linear probe, even in obese patients. In addition, the “ABCDE” technique is simple and produces reliable result even after a quick learning course^34^.

HDP can be detected from the chest radiograph by observing either an absence of downward movement of diaphragm or a paradoxical upward movement during deep inspiration. In an exploratory analysis, we found a positive correlation between the diaphragm elevation on chest radiograph and the DTF. The cut off value of the HDP diagnosis in this study was approximately 29 mm; higher than the 20 mm value used as a more stringent diagnostic criterion in our previous study^13^. It can be useful when determining HDP retrospectively when timely separated images are acquirable.

This study has several limitations. Firstly, the onset and postoperative analgesia were not evaluated. However, there were no cases of block failure or unexpected conversion to general anesthesia in either group. Secondly, patient blinding was not achieved. However, we suspect that the impact of this unblinding on the patient’s breathing effort to be neglectable. Thirdly, the post-blockade assessment of diaphragm and pulmonary function test were performed not during the operation but postoperatively, which may have caused underestimation of HDP. However, considering that the majority of surgeries were concluded within 80 min, unlike the overwhelming duration of local anesthetic in our protocol, the effects should also be negligible. Additionally, in our previous study, HDP was well observed in chest radiographs taken after surgery^13^. Fourth, although B-mode this study was confined to the Korean patients, who are relatively smaller in physique compared to the Western ethnicity. However, we believe that the principal trend of HDP incidence would still be maintained regardless of the population. Fifth, five patients in group C received supraclavicular block due to failure of visualizing costoclavicular space. We found that it is not always easy to acquire optimal image of neural structure in costoclavicular space in patients with thick subcutaneous tissue. Considering the demographic data of other studies using costocalavicular approach (majority of the participants were non-obese), we consider this problem as a common issue^2,3,22^. Future study regarding this issue is warranted. Lastly, all blockades were performed by a single anesthesiologist. Therefore, the results could have depended on the anesthesiologist’s experience and skill. Therefore, caution is needed when generalizing our results. Still, because of the relatively simple and consistent anatomy of costoclavicular space^2^, we believe the occurrence of HDP due to technical aspect is minimal.

In conclusion, costoclavicular block can reduce the incidence of HDP compared to supraclavicular block. Costoclavicular block also has less impact on pulmonary function and should be considered as alternate choice for BPB.

## Supplementary Information

Supplementary Information.

## References

[1] Sala-Blanch X, Reina MA, Pangthipampai P, Karmakar MK (2016). Anatomic basis for brachial plexus block at the costoclavicular space: A cadaver anatomic study. Reg. Anesth. Pain Med..

[2] Li JW, Songthamwat B, Samy W, Sala-Blanch X, Karmakar MK (2017). Ultrasound-guided costoclavicular brachial plexus block: Sonoanatomy, technique, and block dynamics. Reg Anesth Pain Med.

[3] Songthamwat B, Karmakar MK, Li JW, Samy W, Mok LYH (2018). Ultrasound-guided infraclavicular brachial plexus block: Prospective randomized comparison of the lateral sagittal and costoclavicular approach. Reg. Anesth. Pain Med..

[4] Aliste J (2019). Randomized comparison between interscalene and costoclavicular blocks for arthroscopic shoulder surgery. Reg. Anesth. Pain Med..

[5] Garcia-Vitoria C, Vizuete J, Lopez Navarro AM, Bosch M (2017). Costoclavicular space: A reliable gate for continuous regional anesthesia catheter insertion. Anesthesiology.

[6] Karmakar MK, Sala-Blanch X, Songthamwat B, Tsui BC (2015). Benefits of the costoclavicular space for ultrasound-guided infraclavicular brachial plexus block: Description of a costoclavicular approach. Reg. Anesth. Pain Med..

[7] Kim BG (2017). A comparison of ultrasound-guided interscalene and supraclavicular blocks for post-operative analgesia after shoulder surgery. Acta Anaesthesiol. Scand..

[8] Ryu T, Kil BT, Kim JH (2015). Comparison between ultrasound-guided supraclavicular and interscalene brachial plexus blocks in patients undergoing arthroscopic shoulder surgery: A prospective, randomized, parallel study. Medicine (Baltimore).

[9] Gentili ME, Deleuze A, Estèbe JP, Lebourg M, Ecoffey C (2002). Severe respiratory failure after infraclavicular block with 075% ropivacaine: A case report. J. Clin. Anesth..

[10] Kessler J, Schafhalter-Zoppoth I, Gray AT (2008). An ultrasound study of the phrenic nerve in the posterior cervical triangle: Implications for the interscalene brachial plexus block. Reg. Anesth. Pain Med..

[11] Nieuwveld D (2017). Medial approach of ultrasound-guided costoclavicular plexus block and its effects on regional perfussion. Rev. Esp. Anestesiol. Reanim..

[12] Leurcharusmee P (2017). A randomized comparison between costoclavicular and paracoracoid ultrasound-guided infraclavicular block for upper limb surgery. Can. J. Anaesth..

[13] Oh C (2020). Costoclavicular brachial plexus block reduces hemidiaphragmatic paralysis more than supraclavicular brachial plexus block: Retrospective, propensity score matched cohort study. Korean J. Pain.

[14] Harris PA (2019). The REDCap consortium: Building an international community of software platform partners. J. Biomed. Inform..

[15] Moher D (2010). CONSORT 2010 explanation and elaboration: Updated guidelines for reporting parallel group randomised trials. BMJ.

[16] Soares LG, Brull R, Lai J, Chan VW (2007). Eight ball, corner pocket: The optimal needle position for ultrasound-guided supraclavicular block. Reg. Anesth. Pain Med..

[17] Morfey DH, Brull R (2009). Finding the corner pocket: Landmarks in ultrasound-guided supraclavicular block. Anaesthesia.

[18] Tsui JJ, Tsui BC (2016). A novel systematic ABC approach to diaphragmatic evaluation (ABCDE). Can. J. Anaesth..

[19] Boon AJ (2013). Two-dimensional ultrasound imaging of the diaphragm: Quantitative values in normal subjects. Muscle Nerve.

[20] Petrar SD, Seltenrich ME, Head SJ, Schwarz SK (2015). Hemidiaphragmatic paralysis following ultrasound-guided supraclavicular versus infraclavicular brachial plexus blockade: A randomized clinical trial. Reg. Anesth. Pain Med..

[21] R Core Team. *R: A Language and Environment for Statistical Computing*. (R Foundation for Statistical Computing, 2020). https://www.R-project.org/. Accessed 8 July 2021.

[22] Sivashanmugam T, Maurya I, Kumar N, Karmakar MK (2019). Ipsilateral hemidiaphragmatic paresis after a supraclavicular and costoclavicular brachial plexus block: A randomised observer blinded study. Eur. J. Anaesthesiol..

[23] Renes SH, Spoormans HH, Gielen MJ, Rettig HC, van Geffen GJ (2009). Hemidiaphragmatic paresis can be avoided in ultrasound-guided supraclavicular brachial plexus block. Reg. Anesth. Pain Med..

[24] Kang RA, Chung YH, Ko JS, Yang MK, Choi DH (2018). Reduced hemidiaphragmatic paresis with a "corner pocket" technique for supraclavicular brachial plexus block: Single-center, observer-blinded, randomized controlled trial. Reg. Anesth. Pain Med..

[25] Gottesman E, McCool FD (1997). Ultrasound evaluation of the paralyzed diaphragm. Am. J. Respir. Crit. Care Med..

[26] Mak PHK, Irwin MG, Ooi CGC, Chow BFM (2001). Incidence of diaphragmatic paralysis following supraclavicular brachial plexus block and its effect on pulmonary function. Anaesthesia.

[27] Golarz SR, White JM (2020). Anatomic variation of the phrenic nerve and brachial plexus encountered during 100 supraclavicular decompressions for neurogenic thoracic outlet syndrome with associated postoperative neurologic complications. Ann. Vasc. Surg..

[28] Loukas M, Kinsella CR, Louis RG, Gandhi S, Curry B (2006). Surgical anatomy of the accessory phrenic nerve. Ann. Thorac. Surg..

[29] Bigeleisen PE (2003). Anatomical variations of the phrenic nerve and its clinical implication for supraclavicular block. Br. J. Anaesth..

[30] Naik LY, Sondekoppam RV, Jenkin Tsui J, Tsui BC (2016). An ultrasound-guided ABCDE approach with a sniff test to evaluate diaphragmatic function without acoustic windows. Can. J. Anaesth..

[31] Tsui BC, Tsui J (2016). ABC diaphragmatic evaluation for neonates. Paediatr. Anaesth..

[32] El-Boghdadly K, Chin KJ, Chan VWS (2017). Phrenic nerve palsy and regional anesthesia for shoulder surgery: Anatomical, physiologic, and clinical considerations. Anesthesiology.

[33] Testa A (2011). Ultrasound M-mode assessment of diaphragmatic kinetics by anterior transverse scanning in healthy subjects. Ultrasound Med. Biol..

[34] Khurana J, Gartner SC, Naik L, Tsui BCH (2018). Ultrasound identification of diaphragm by novices using ABCDE technique. Reg. Anesth. Pain Med..

