# Author Correction: Hemidiaphragmatic paralysis following costoclavicular versus supraclavicular brachial plexus block: a randomized controlled trial

**DOI:** 10.1038/s41598-021-00054-7

**Published:** 2021-10-12

**Authors:** Boohwi Hong, Soomin Lee, Chahyun Oh, Seyeon Park, Hyun Rhim, Kuhee Jeong, Woosuk Chung, Sunyeul Lee, ChaeSeong Lim, Yong-Sup Shin

**Affiliations:** 1 Department of Anesthesiology and Pain Medicine, Chungnam National University Hospital, 282 Munhwa-ro, Jung-gu, Daejeon, 35015 Korea; 2 Department of Anesthesiology and Pain Medicine, College of Medicine, Chungnam National University, Daejeon, Republic of Korea; 3 Department of Nursing, Chungnam National University, Daejeon, Republic of Korea

Correction to: *Scientific Reports* 10.1038/s41598-021-97843-x, published online 21 September 2021

The original version of this Article contained an error in Affiliation 3, which was incorrectly given as ‘Department of Nursing, Health Institute of Technology, Daejeon, Republic of Korea.’

The correct affiliation is listed below:

Department of Nursing, Chungnam National University, Daejeon, Republic of Korea.

The original Article and accompanying Supplementary Information file has been corrected.

