# Supplementary material for: Hemidiaphragmatic paralysis following costoclavicular versus supraclavicular brachial plexus block: a randomized controlled trial
Source: Sci Rep. 2021 Sep 21;11:18749. doi: 10.1038/s41598-021-97843-x (PMC8455610; doi:10.1038/s41598-021-97843-x)
Supplement: Supplementary file 1 — Supplementary Information. [file 41598_2021_97843_MOESM1_ESM.docx]

**Hemidiaphragmatic Paralysis Following Costoclavicular Versus Supraclavicular Brachial Plexus Block: A Randomized Controlled Trial**

Boohwi Hong, M.D., Ph.D.^1, 2 #^, Soomin Lee, M.D., Ph.D.^1 #^, Chahyun Oh, M.D.^2^, Seyeon Park, Ph.D., R.N.^3^, Hyun Rhim, M.D^1^., Kuhee Jeong, M.D.^1^, Woosuk Chung, M.D., Ph.D.^1,2^, Sunyeul Lee, M.D., Ph.D.^1,2^, ChaeSeong Lim, M.D., Ph.D.^1,2^, Yong-Sup Shin, M.D., Ph.D.^1,2^

^1^ Department of Anesthesiology and Pain Medicine, Chungnam National University Hospital, Daejeon, Republic of Korea

^2^ Department of Anesthesiology and Pain Medicine, College of Medicine, Chungnam National University, Daejeon, Republic of Korea

^3^ Department of Nursing, Chungnam National University, Daejeon, Republic of Korea

# First author, contributed equally

**Corresponding Author**: Yong-Sup Shin, M.D., Ph.D.

**Mailing address**: Department of Anesthesiology and Pain Medicine, Chungnam National University Hospital

282 Munhwa-ro, Jung-gu, Daejeon 35015, Korea.

**Tel**: +82-42-280-7840

**Fax**: +82-42-280-7968

**E-mail**: [ysshin@cnu.ac.kr](mailto:ysshin@cnu.ac.kr)

Supplement Table 1. Results stratified by hemidiaphragmatic paralysis

|  | HDP  (n=23) | No HDP  (n=52) | Effect size (95% CI) | P value |
| --- | --- | --- | --- | --- |
| Pre-block DTF (%), median [IQR] | 83.3 [64.5;100.0] | 88.1 [62.7;117.9] | -4.8 [-28.5 to 12.9] | 0.414^a^ |
| Post-block DTF (%), median [IQR] | 7.1 [ 0.0;12.9] | 60.0 [40.0;82.3] | -52.8[-70.6 to -40.7] | <0.001^a^ |
| Δ DTF (%), median [IQR] | 77.8 [61.0;94.9] | 27.1 [ 0.0;50.8] | 50.7[33.3 to 72.8] | <0.001^a^ |
| Δ FVC (L), median [IQR] | 0.5 [ 0.3; 0.9] | 0.2 [-0.1; 0.4] | 0.3 [0.2 to 0.7] | <0.001^a^ |
| Δ FEV1 (L), median [IQR] | 0.7 [ 0.4; 0.8] | 0.2 [ 0.0; 0.4] | 0.5[0.3 to 0.6] | <0.001^a^ |
| Δ PEFR (L/sec), median [IQR] | 1.4 [ 0.7; 1.8] | 0.7 [0.0; 1.8] | 0.7[-0.1 to 1.2] | 0.225 ^a^ |
| Δ FVC (%), median [IQR] | 16.5 [10.6;26.1] | 4.6 [-2.6;11.0] | 11.9[4.6 to 22.1] | <0.001^a^ |
| Δ FEV1 (%), median [IQR] | 24.8 [11.5;31.2] | 7.5 [ 0.9;11.8] | 17.4[6.9 to 24.4] | <0.001^a^ |
| Δ PEFR (%), median [IQR] | 22.6 [10.2;35.8] | 11.8 [-0.3;24.3] | 10.8[-2.4 to 19.6] | 0.097^a^ |

Effect size (median differences) are differences (HDP – No HDP). ^a^ Mann–Whitney *U* test

DTF=diaphragm thickning fraction; HDP=hemidiaphragmatic paralysis (DTF < 20%); FVC= forced vital capacity; FEV1: forced expiratory volume in 1 second; PEFR= peak expiratory flow rate; Δ delta=(pre – post value); CI=confidence interval
